# The informed consent form navigator: a tool for producing readable and compliant consent documents

**DOI:** 10.1017/cts.2022.507

**Published:** 2022-12-01

**Authors:** Jonathan P. Bona, Joseph Utecht, Aaron S. Kemp, Jennifer M. Gan, Alison Caballero, Christopher R. Trudeau, Mathias Brochhausen, Laura James

**Affiliations:** 1 Department of Biomedical Informatics, University of Arkansas for Medical Sciences, Little Rock, AR, USA; 2 Department of Psychiatry, University of Arkansas for Medical Sciences, USA; 3 Center for Health Literacy, University of Arkansas for Medical Sciences, USA; 4 Department of Medical Humanities and Bioethics, University of Arkansas for Medical Sciences, USA; 5 University of Arkansas Little Rock William H. Bowen School of Law, Little Rock, AR, USA; 6 Translational Research Institute, University of Arkansas for Medical Sciences, USA; 7 Department of Pediatrics, University of Arkansas for Medical Sciences, USA

**Keywords:** Informed consent, research informatics, health literacy, health equity, tool development

## Abstract

**Background/Objective:**

Informed consent forms (ICFs) and practices vary widely across institutions. This project expands on previous work at the University of Arkansas for Medical Sciences (UAMS) Center for Health Literacy to develop a plain language ICF template. Our interdisciplinary team of researchers, comprised of biomedical informaticists, health literacy experts, and stakeholders in the Institutional Review Board (IRB) process, has developed the ICF Navigator, a novel tool to facilitate the creation of plain language ICFs that comply with all relevant regulatory requirements.

**Methods:**

Our team first developed requirements for the ICF Navigator tool. The tool was then implemented by a technical team of informaticists and software developers, in consultation with an informed consent legal expert. We developed and formalized a detailed knowledge map modeling regulatory requirements for ICFs, which drives workflows within the tool.

**Results:**

The ICF Navigator is a web-based tool that guides researchers through creating an ICF as they answer questions about their project. The navigator uses those responses to produce a clear and compliant ICF, displaying a real-time preview of the final form as content is added. Versioning and edits can be tracked to facilitate collaborative revisions by the research team and communication with the IRB. The navigator helps guide the creation of study-specific language, ensures compliance with regulatory requirements, and ensures that the resulting ICF is easy to read and understand.

**Conclusion:**

The ICF Navigator is an innovative, customizable, open-source software tool that helps researchers produce custom readable and compliant ICFs for research studies involving human subjects.

## Introduction

The interactive process of obtaining informed consent is critical for human subjects research, and it is often documented by the signing of an informed consent form (ICF), affirming that the subject understands key aspects of the research and the subject’s role in the research and agrees to participate. Due to the complexity of research protocols, frequent use of medical jargon, and mandated compliance with regulatory requirements, ICFs are often long and difficult to read [1]. This can be a major barrier for the inclusion of individuals who may have limited health literacy skills, including individuals from underserved or underrepresented minority populations [2–4]. Accordingly, there is an ethical imperative to improve the overall readability of informed consent documents so that they convey key information about a study in a manner that can be easily understood by all prospective research participants. In the USA, this ethical goal has been translated into a regulatory requirement as the Revised Common Rule, which now requires that consent forms be written in a manner that is readily understandable to all prospective research participants [5,6].

The Center for Health Literacy at the University of Arkansas for Medical sciences (UAMS) previously developed a plain language informed consent form (PL ICF) template, which is currently publicly available to all researchers as a Microsoft Word document (https://healthliteracy.uams.edu/health-literacy-research/resources/). Formal evaluations of this template have demonstrated that ICFs created using the PL ICF template had an average readability score at the 7th-grade reading level, while those created without using a template had an average readability at the 10th-grade level [6,7]. While these templates improved readability, their use requires researchers to read through the instructions, answer relevant questions, and then remember to delete the questions, instructions, and unrelated sections from the form before use. In reviewing ICFs developed using the PL ICF template, our team noted that many Institutional Review Board (IRB)-required elements were removed in error, researchers often failed to remove instructions or sections that were not relevant to their study, and the investigator-entered language was often written at an advanced reading level, making it inaccessible to some prospective participants [6,7].

To address these barriers, a team of researchers supported by the Translational Research Institute, our Clinical and Translational Science Award (CTSA), created the ICF Navigator, a web-based software tool that guides researchers through the interactive process of creating an ICF as they answer a series of questions about their research. This manuscript presents and discusses the ICF Navigator tool, which is designed to (1) guide researchers through the creation of study-specific ICF language, (2) ensure compliance with all pertinent regulatory requirements, and (3) produce an ICF written in plain language that will be easy to understand, particularly by individuals at risk for limited health literacy skills.

## Materials and Methods

The ICF Navigator development team was comprised of interdisciplinary experts including: clinical trialists; biomedical informaticists who specialize in knowledge representation, ontologies, and natural language processing; and others with expertise in software development, research ethics, health literacy, health education, and plain language writing. The initial development of the navigator’s questions and decision tree were developed by a legal expert in informed consent for clinical research.

The resulting tool, the ICF Navigator, is a custom-built application implemented in Python [8] and Javascript [9]. It uses the Postgres relational database system [10] to store data on its back end. The initial configuration of the ICF Navigator includes 93 questions, with survey logic to dynamically adjust and reduce the number of questions a user must answer based on properties of the research. Underlying this application is a detailed knowledge map that models regulatory requirements for ICFs and is used to drive customized survey logic that guides the user through their use of the system.

This system uses exemplary text from the plain language ICF template developed by experts at the UAMS Center for Health Literacy. This exemplary text is used to automatically populate key sections of the ICF based on the user’s answers to questions presented by the ICF Navigator. These elements are described in more detail in the following subsections.

### Knowledge Map for Survey Logic

The ICF Navigator’s questionnaire logic was built to optimize the user’s interactions with the tool by eliminating input of redundant or unapplicable information, based on detailed modeling of regulatory requirements for ICFs in the form of a knowledge map. This knowledge map has two important functions: (1) during initial development it helped to ensure that the navigator tool covered all regulatory possibilities and requirements, and (2) it has been integrated into the tool to direct the flow of the navigator’s core question and answer functionality.

The detailed knowledge map captured these requirements and dependencies and was used to guide the navigator’s question and answer functionality. The knowledge map captured all the requirements of the Revised Common Rule as well as institution-specific requirements. Under the Revised Common Rule, there are up to 18 required elements that must be included in a consent form depending on the nature of the specific study [11].

Not every study’s ICF will need all of these elements. For example, some requirements apply only to studies involving biospecimens. The tool assists researchers in selecting only those elements that are required in their study.

To accomplish this, the team used the Informed Consent Ontology (ICO) [12,13] to help categorize the requirements into broad categorical areas of the knowledge map. This resulted in 10 core categories of questions in the knowledge map:

1. Introductory Questions
2. Key Information
3. About the Study
4. Study Removal
5. Costs & Compensation
6. Benefits & Alternatives
7. Results, Impact, & Disclosure
8. Risks & Side Effects
9. Biospecimens & Future Research
10. Confidentiality

This categorization was used to optimize the user experience for researchers using the navigator. An early goal for the system was to have related questions flow naturally from one to the next rather than jump around from topic to topic.

**Fig. 1.**
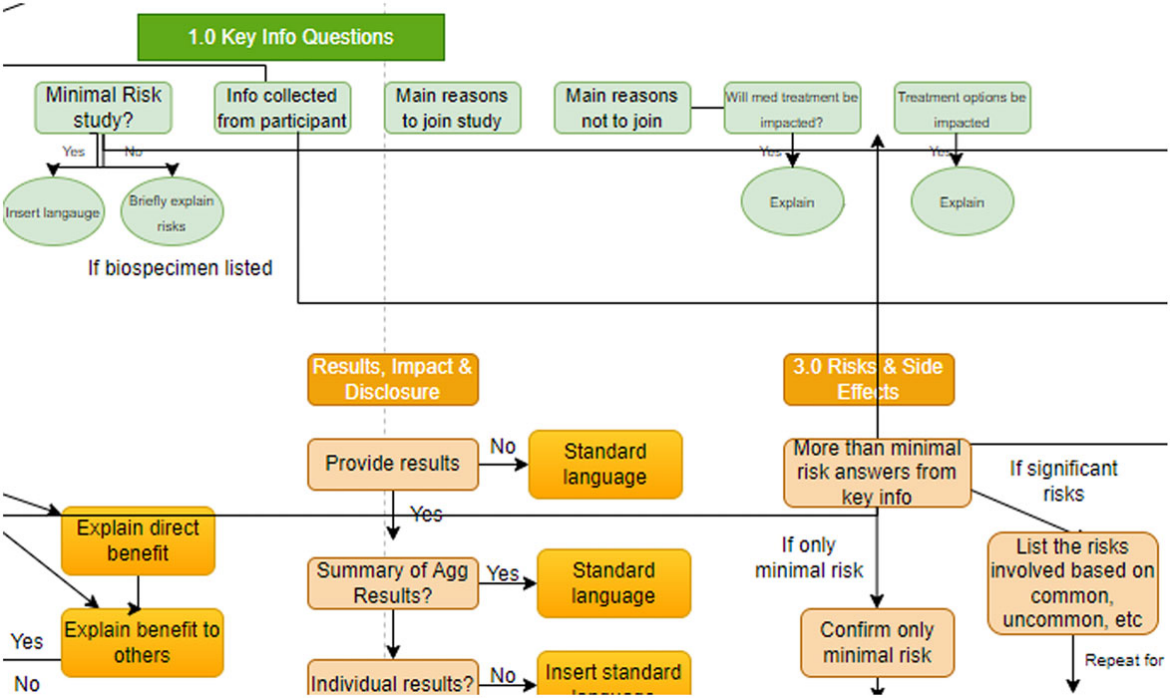
An excerpt of the knowledge map. Questions for major sections of the form (orange) in some cases depend on Key Information (green), which is collected first.

Using this knowledge map, the team developed 93 context-dependent questions to guide researchers through specific sections of the consent form, ensuring that all required components of the Revised Common Rule were represented. Fig. 1 shows an excerpt of the knowledge map, including areas related to the *Key Information*, *Results, Impact & Disclosure*, and *Risks & Side Effects* core categories.

Within the ICF Navigator system, questions are managed by customizable survey logic that dynamically adjusts to only ask questions relevant to the study. For example, when a user indicates early in the process that the study does not involve biospecimens, then the system automatically skips subsequent sections of questions about collection, storage, and sharing of biospecimens.

This survey logic helps to tailor what the researcher sees – and needs to respond to – when creating an ICF for their specific study. This is intended to reduce the user’s cognitive burden and eliminate the errors typically made when using print-based templates.

This question set was also used to guide the mapping of previously configured PL ICF template language based on specific answers provided to many of the questions in the navigator. For example, if a researcher is conducting a double-blind study with a placebo, the navigator inserts plain language explanations of what those terms mean in the “About the Study” section of the ICF. This prepared language was developed by plain language experts at the UAMS Center for Health Literacy with review and input from our IRB. Having this language preapproved will help ensure a smooth IRB review process for researchers using the navigator.

After the initial prototype is complete, the team will hold future focus group sessions to test the output of the ICF Navigator, including participants with limited health literacy. Feedback from this focus group will be used to further ensure that the boilerplate language is easy to understand and use.

### Implementation of the ICF Navigator Application

In order to use the knowledge map and associated questions required to produce a consent form, we created a custom-built survey application that is tailored to this use within the ICF Navigator. Prior to developing this, we reviewed available generic survey applications and found they were lacking key features required for the ICF Navigator. These important features, which we built into the ICF Navigator survey application, included the ability to view sample output of the consent form in real time as questions are answered by the user, rich sharing features to allow multiple collaborators to contribute to a single form, and the flexibility to use prepared text that can be edited in the same way as newly added text can be edited by the user.

In order for a tool like the ICF Navigator to guide the user through creating a complete and compliant ICF, it must present a fairly large number of questions, though in a typical interaction the user will not see all 93 questions present in the system because the tool tries to show only those questions relevant to a study. As these questions are answered through interaction with the tool, it displays a real-time preview of the output form that is being generated. This feature is intended to increase completion rate by showing users their immediate progress through the survey, and it also encourages users to enter responses that flow naturally with adjacent sections of the output form. Because the final output is often a lengthy complete ICF, the real-time preview that is generated as the user enters information displays only a formatted excerpt showing the section of the form most relevant to the questions that are currently being asked/answered.

We identified and implemented six major types of question formats that are required within the ICF Navigator; yes/no, yes/no/explain, multi-select, text list, text response, and contact information.

The *yes/no question* and *multi select questions* are used to drive logic in the form. For example, by asking early in the questionnaire about properties of the study (e.g., does the study involve biospecimens), relevant subsequent sections of the questionnaire are disabled or enabled based on the response.

The *yes/no question* type is also used where sections of prewritten text may need to be inserted. After a user selects yes or no to one of these questions, the relevant prewritten text appears in the form preview and will appear in the final form. For example, if the researcher answers “No” to the question “Can a participant involuntarily be removed from the study?”, then the final ICF form will include the statement “You cannot be removed from the study if you want to continue.”

Another type of common question is the *text list question*, where the user is asked to provide a list of responses that will be displayed on the form as a bulleted list. For example, if the researcher answers “Yes” to the involuntary removal question, they are prompted to select possible reasons for the removal of a participant. In this case, the final form will then contain the statement “The head researcher can take you out of the study if,” followed by a list of possible reasons for involuntary removal.

Many sections of the form display information about the primary investigator or other individuals on the research team, often in the context of contacting a person with study questions or concerns. To keep the names and contact information easy to read, the *contact information question* breaks apart the titles, address, and names of a person into separate fields.

Finally, the *text question input type* is used when the user response text will be directly inserted into a section of the form. Prompts to the user within the navigator remind the user that their typed content will appear as written in the output, and that they should write in full sentences using plain language.

Question dependency is an important feature within the ICF Navigator, as many questions are not relevant to all studies. The knowledge map introduced above, which encodes information about regulatory requirements and workflow, is used by the ICF Navigator to accomplish this. Questions are grouped into major sections, determining which the part of the output form to display to the user, as well as question groups, which are displayed or hidden together based on a simple logic statement. An early example section is Key Information. Questions in this section serve the function of populating the first pages of the consent form but also determine dependency for entire later sections. All of the questions and logic are stored in the database and can be modified through an administrator interface without the involvement of a software developer.

The ICF Navigator administrator interface was designed to allow authorized users without software development expertise to modify the application to suit the needs of an institution. This allows for institution-specific customizations, as well as modifications that may be necessitated by future regulatory changes. From the administrator interface, questions can be added, changed, or removed. The order and grouping of sections and question groups can be changed. Prepared text and the layout of the output form can also be edited to accommodate institution-specific requirements.

To aid in producing readable and understandable text within ICFs, the system provides real-time feedback on free text as users enter it, providing a calculated readability score and suggestions to improve readability. This feature is discussed in more detail below.

The ICF Navigator also provides tools for managing a library of ICFs in various stages of development. This includes an association between individual forms and the studies for which they are produced, as well as the ability within the system for a researcher to invite collaborators and provide them with permission to view or make changes to a study’s form.

### Real-time Readability Feedback

While the tool includes a large bank of prepared text to support research across a wide range of topics, many sections of ICFs may require study-specific language entered by the researcher building the form. In those areas, the navigator will prompt the researcher to enter their own text but provides instructional text and examples of content to include. An advantage of in-tool guidance over print templates is that the researcher does not need to remember to delete instructions as they would if using print templates – the navigator only inserts what researchers themselves write into the user interface. To promote the use of readable and understandable text in these fields, in pursuit of compliance with the Revised Common Rule, the ICF Navigator system provides real-time feedback on the readability.

The system calculates readability scores for user-entered text using a combination of scores obtained from Flesch–Kincaid [14], SMOG [15], and Coleman–Liau [16] formulas. Each of these metrics is determined by three variables: sentence count, word count, and syllable count. Because these metrics vary in how they score short writing samples, we used a combination of these scores to increase the consistency of scoring short text responses. On fields that accept user-entered free text, the ICF Navigator presents a grade-level readability score and provides specific feedback on how input can be improved based on simple rules applied to the user-entered text. In particular, this feedback notes the number of long (more than 15 words) sentences, and words with three or more syllables. For individual question responses, real-time feedback is shown to the user as they type.

### Technical Implementation

The ICF tool was built primarily using the open-source Python library Django [17]. This library handles web requests, manages the database, and provides the administration interface. Some user interface features were implemented using Stimulus.js [18] to allow for JavaScript code to manage features such as question dependency, refreshing real-time previews of the output form, and readability scoring for free text fields. Text questions were implemented using the Trix editor [19] to allow the user some amount of familiar rich text formatting, such as adding bold or italics. The ICF Navigator source code has been published as open source, with a permissive license, and is available on GitHub [20].

## Results

We have built and deployed the ICF Navigator tool and conducted preliminary usability testing. The below subsections illustrate and describe the tool’s functionality, show part of a completed ICF generated using the tool, and detail the usability evaluation we have conducted.

### ICF Navigator Interface and Workflow

Upon logging in to the ICF Navigator, the user is presented with a list of projects and forms they can access. A user has access to a project if they initially created the project in the system, or if someone else on the user’s research team has created a project and added them to it. The project titles and information about when each project was last changed are presented in a chronologically ordered list, facilitating quick access to more recent work.

The user can initiate a new project to build a new ICF by entering a short title and pressing a button labeled “Start.”

**Fig. 2.**
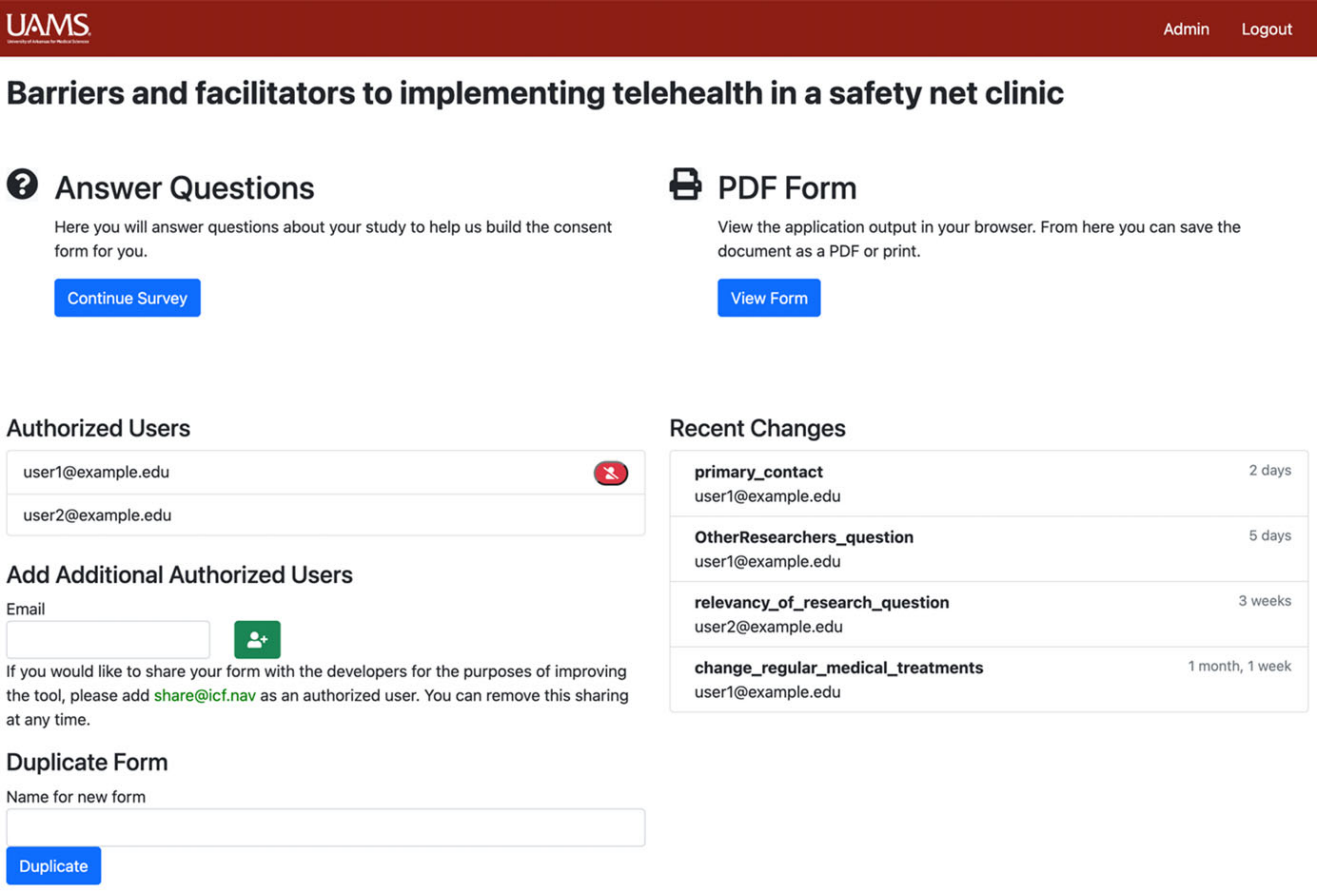
Informed consent form project landing page.

Upon selecting an existing project or initiating a new project, the user enters a project landing page, which prominently displays the project title, as shown in Fig. 2. On this page, the user may view, add, or remove authorized users, as shown at the left. This page also tracks and displays recent changes that have been made to the form, listing both the section changed and the email address of the user who made the change. The recent changes area lists usernames and section names for recent edits to the form. To populate the ICF with content, the user selects “Continue Survey” in the upper left portion of the page. The “View Form” button on the right-hand side is used to view, download, or print a PDF of the form when it is finished.

**Fig. 3.**
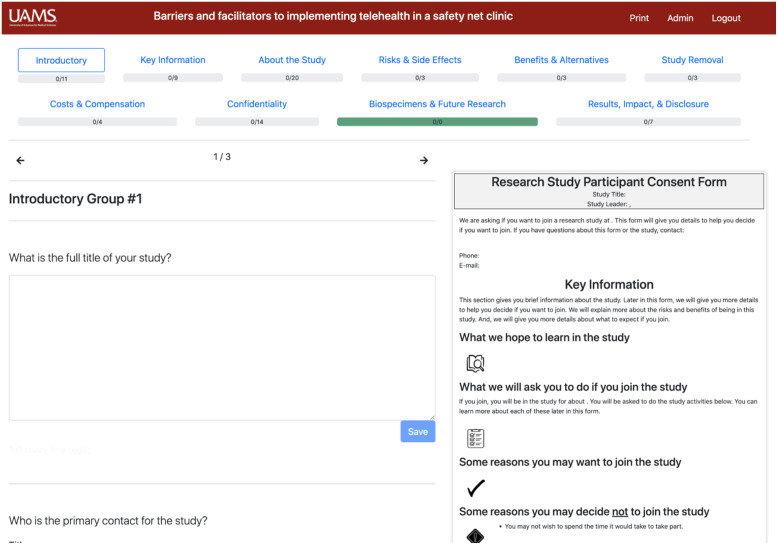
Informed consent form survey main page.

Fig. 3 shows the main page that a researcher uses to populate an ICF. This is the view for a new project that has not had any information entered yet. At the top of this page are the survey’s major section headings: Introductory, Key Information, About the Study, Risks & Side Effects, and so on. As the user completes information entry for each section, the user can navigate directly to a section by clicking on the section’s name. Progress bars illustrate the relative completion of each section.

On the right-hand side of the ICF main page (Fig. 3), a real-time preview shows the relevant section’s appearance as it would appear on the final ICF.

**Fig. 4.**
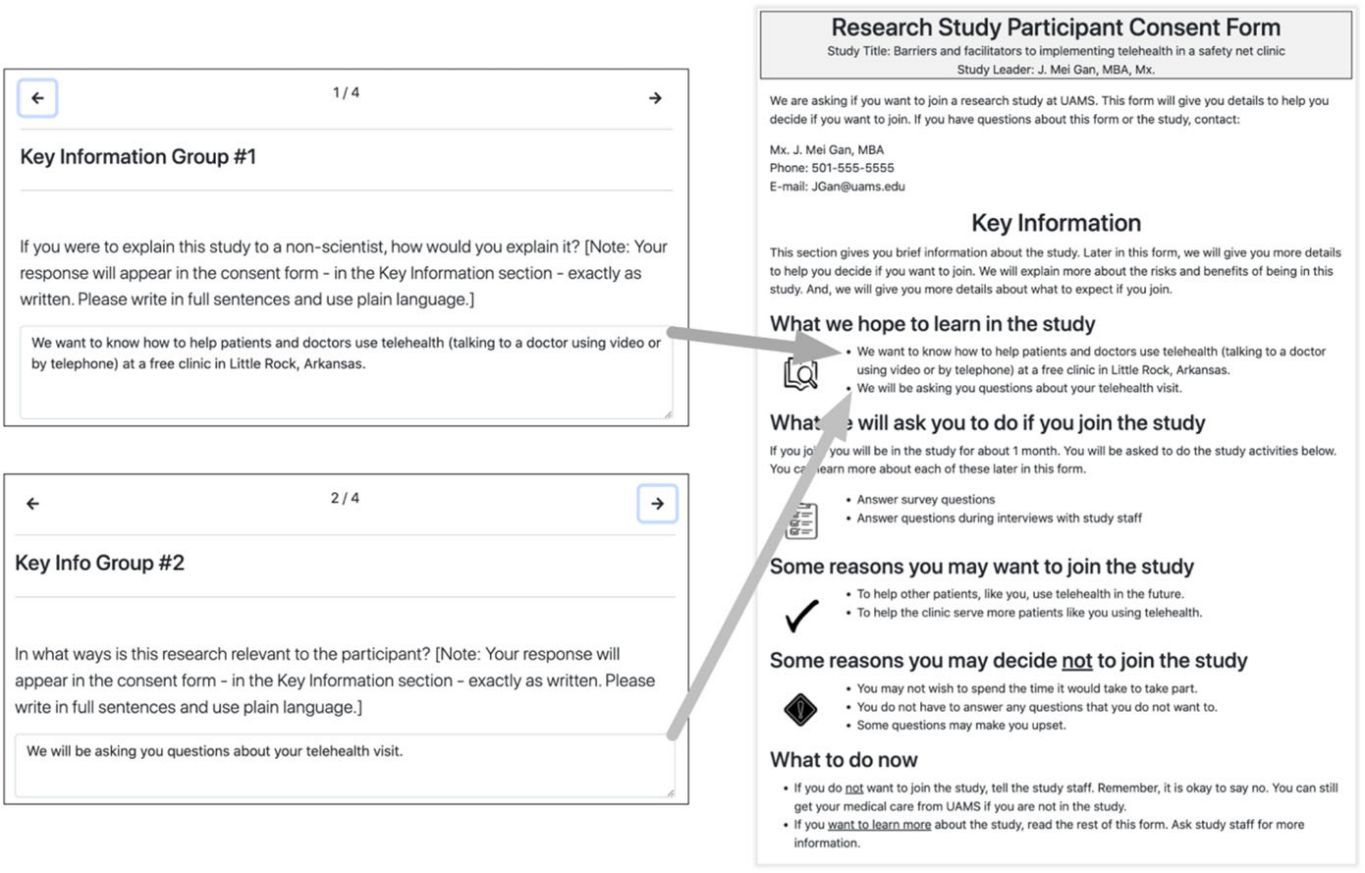
Answers to two Key Information questions combined in the final form preview.

Fig. 4 shows how the user input answers to two Key Information Group questions are combined in the preview of the final form. The first question prompts the user to explain the study for a non-scientist audience, and the second asks the user to explain how the research is relevant to a participant. These are combined in the form preview, and in the final form, in the Key Information section under the heading “What we hope to learn in the study.”

The progress bar displayed in navigator section headings, which is visible at the top of Fig. 3, adjusts dynamically as the user enters information. The number of expected elements per section may change depending on answers in other sections, as discussed in earlier sections detailing the knowledge map and survey logic underlying the ICF Navigator tool. For example, the Biospecimens & Future Research section initially shows that 0 out of 0 elements have been completed. If the user indicates in the Introductory section that the research project involves Biospecimen Collection, then the Biospecimens & Future Research heading is automatically updated to show that 0 out of 4 required elements have been completed in that section, reflecting that there are four elements that must be completed for studies involving biospecimens.

An ICF need not be completed within a single session. Rather, the user or users editing a project may log out of the ICF Navigator at any time and return later to continue creating a form. When the researcher or research team has finished creating a form in the ICF Navigator, a PDF version of the resulting form can be accessed via the “PDF Form” section of the project landing page, as shown in Fig. 2. The resulting file can be printed directly from the tool or downloaded and distributed as a PDF.

### Final Output Form

**Fig. 5.**
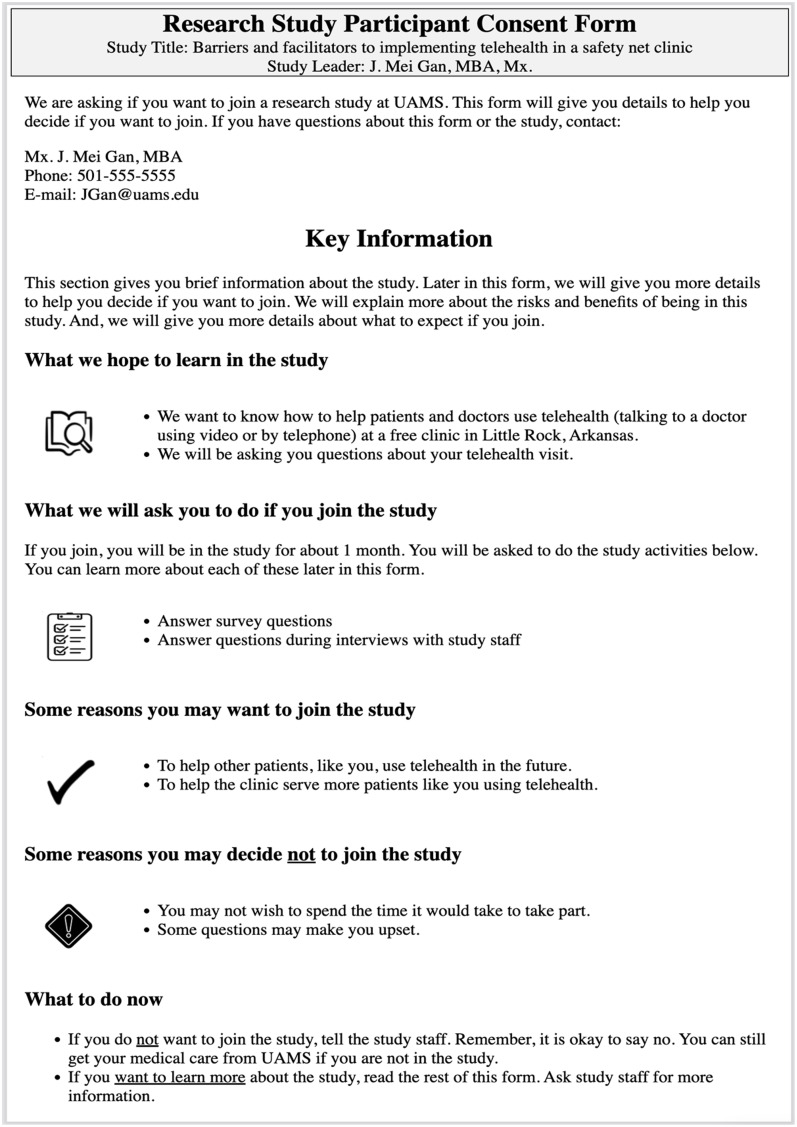
First page of a completed navigator-generated informed consent form, showing contact information and other introductory and key information.

The final output of this process is a readable, printable ICF document aligned with the plain language templates developed by the UAMS Center for Health Literacy and populated with information entered by the research team using the ICF Navigator tool. A typical ICF document is several pages long. An example of the full first page of a form containing contact and other introductory and key information is shown in Fig. 5.

### Preliminary Usability Testing

Initial reactions and feedback on the design of the ICF Navigator were obtained from a small group of local stakeholders, including the Director of the UAMS IRB, TRI faculty and staff, and a research ethicist, following a brief walk-through of the online interface as it was being developed. This initial feedback served to guide further development and refinement of the interface. Once a working prototype was complete, preliminary usability testing of the ICF Navigator was conducted among five seasoned researchers from TRI, Arkansas Children’s Hospital, the Psychiatric Research Institute, and the Winthrop P. Rockefeller Cancer Institute, each with experience in clinical study management or informed consent procedures. These users recreated a total of seven unique ICFs using the ICF Navigator. Each tester provided written feedback on perceived usability of the interface and suggested improvements. Their suggested improvements have already been implemented in the ICF Navigator tool. As an example, the progress bars accompanying section headings shown and discussed earlier were not part of the initial prototype but were included as the result of user feedback from this initial testing. A number of minor software bugs in the initial prototype were also identified and fixed based on this testing.

## Discussion and Future Work

The ICF Navigator is a software tool to assist researchers in creating plain language ICFs that meet regulatory requirements, leveraging a set of integrated questions which guide the researcher through the creation of an ICF. We have completed and deployed the first release of this tool and conducted preliminary usability testing.

Our next step in evaluating the ICF Navigator, currently underway, is to test output ICFs with a community audience focus group inclusive of individuals with limited health literacy. This will allow us to test that the ICFs produced by the tool are optimally readable, understandable, and actionable and may generate ideas for further improvement.

As future work, we will integrate natural language processing and machine learning approaches to working with free text. This will allow the tool to provide more sophisticated real-time guidance on readability of entered text and allow use of more context-specific language based on the researcher’s answers to questions about their study. Additional future work, as described in more detail below, will include the development and integration of electronic consent (eConsent) capabilities, further integration with the ICO, and comprehensive usability evaluations across a variety of intended end users at multiple CTSA sites. Once these additional steps have been completed, the ICF Navigator will be made freely available to all collaborating CTSA sites and eventually to any institution interested in facilitating the creation of plain language ICFs that are designed to meet the diverse needs of researchers at their site.

While the ICF Navigator software itself has been developed to be language-agnostic and can be configured to use templates for developing forms in languages other than English, we have worked with English language forms only in the initial development. Another area of future work is to fully test the system’s multilanguage support.

### Enhanced Ontology Support and Electronic Consent

In any effort to integrate data from heterogeneous sources and provide the ability to identify cohorts across multiple different datasets or institutions, harmonization and integration of data become crucial. For example, if two or more sites using the ICF Navigator tool would like to share information with each other about how many studies at each are or are not collecting biospecimens, the use of a standardized representation of this information will be valuable.

Ontologies are one key strategy for data integration and harmonization, allowing querying data from heterogeneous sources [21,22]. Many ontologies catering to those needs, as, for example, the ICO [12,13], were initially developed to provide a coherent representation of informed consent specifically for but not restricted to the eConsent process. ICO is an OWL ontology and uses Basic Formal Ontology [23] as its upper ontology. It is developed and maintained in accordance with the OBO Foundry principles and workflows [24,25]. Subsequently, ICO was harmonized with a number of related ontologies, namely the Document Act Ontology (d-acts) [26,27], Data Use Ontology (DUO) [28], and Regulatory Basis for Informed Consent Ontology (RUBRIC) [29]. ICO is a reference ontology, and it represents processes, information entities (e.g., legal documents), and realizable entities (e.g., roles and obligations) that are relevant to informed consent [21].

The objective of Amith *et al*. [21] is to represent and operationalize permissions created through the informed consent process based on information contained in the ICFs. In their research, this team used a newly created version of ICO that contained permissions [21], such as the permission to recontact or the permission to perform a biopsy. Using the Semantic Web Rule Language (SWRL) [30], they were able to derive information about permissions from ICFs in one study. This is an encouraging result, and we plan to demonstrate usability on more generalizable and diverse use cases. We also hold that to provide a comprehensive representation of legal entities created by the informed consent process, the ability to present permissions is not sufficient. The representation of obligations also needs to be supported. Members of our team and the curation team of the d-acts ontology have recently extended d-acts [31] to represent the four basic components of rights (claim, power, privilege, and immunity), and their opposites (duty, disability, and liability) according to the legal theory of Hohfeld [32]. Our plan is to use ICO, enriched by novel development in d-acts, to represent and query diverse informed consent information for the legal rights and obligations created in the informed consent process.

We hypothesize that furthering ICO support for the ICF Navigator tool will foster two types of benefits:

- Ontology-based support of ICF creation by enabling easy querying and lookup of similar studies to consider reuse of ICF text blocks, the use of ICO as a controlled vocabulary providing definitions and examples for informed consent-related terms, and consistency check of study description using informed consent processes of similar studies.
- Once the studies are conducted and the data are made available, the ICFs annotated with ICO will facilitate searching for cohorts of data with specific ICF characteristics and thus facilitate secondary use.

While the ability to manage the consenting process or obtain consents electronically is not currently part of the ICF Navigator, our next phase of development will explore using forms built in the ICF Navigator for eConsent. Although initially envisioned as a tool to help researchers create plain language ICFs, the ability to conduct an entire consent process using a digital interface would enable presentation of multimedia content that would not be possible with a printed paper ICF. As such, the integration of eConsent capabilities is anticipated to extend the overall functionality of the ICF Navigator to also include the creation and IRB review of multimedia content, such as brief videos to explain study procedures, graphics that may make complex concepts or study designs easier to convey, and audio files of ICF content for individuals with sight or literacy limitations.

The recent global pandemic has also buoyed interest in eConsent capabilities as a means of facilitating remote participation in telehealth-enabled research studies. This increased interest has led to a number of commercial software applications for eConsent, as well as the addition of eConsent modules to freely available research tools such as REDCap [33]. Notably missing from these prior implementations for eConsent, however, is system-wide integration with existing ontologies to support the management and tracking of digitally acquired consent processes and electronic signatures that comply with regulatory requirements, such as Food and Drug Administration’s Code of Federal Regulations (CFR), Title 21, Part 11. Accordingly, the eConsent capabilities within the ICF Navigator will be designed to leverage the ICO, as described above, and facilitate compliance with 21 CFR, Part 11.

### Comprehensive Usability Testing

During the final phases of ICF Navigator development, comprehensive usability evaluations will be conducted among a multidisciplinary pool of intended end users using qualitative “think-aloud” protocols and a quantitative user interaction satisfaction scale. As traditionally implemented, think-aloud protocols require the prospective end user to verbalize their inner dialog while concurrently interacting with a digital interface to perform various tasks. These methods are commonly applied to elicit insights into the thought processes of the user that can be difficult to ascertain from mere observation and are regarded by many to be the “gold-standard” of usability testing [34,35]. Comprehensive usability testing of the ICF Navigator will also employ quantitative usability/acceptability assessment instruments, such as the Questionnaire for User Interaction Satisfaction (QUIS) [36,37]. The QUIS contains a demographic questionnaire, a measure of overall system satisfaction along six scales, and hierarchically organized measures of 11 specific interface factors. Each area measures the users' overall satisfaction with that facet of the interface, as well as the factors that make up that facet, on a nine-point scale [36,37].

## Conclusion

The ICF Navigator is an innovative, customizable, open-source software tool that helps researchers produce ICFs for research studies involving human subjects. It helps guide the creation of study-specific language, ensures compliance with regulatory requirements, and ensures that the resulting ICF is easy to read and understand.
